# Comparison of experiences and preferences following non-invasive cardiovascular risk procedures: a cross-sectional survey in participants with and without diabetes mellitus

**DOI:** 10.1007/s40200-022-00996-3

**Published:** 2022-03-14

**Authors:** Anchal Lal, Neha Dave, Samia Kazi, Paul Mitchell, Aravinda Thiagalingam

**Affiliations:** 1 Department of Cardiology, Room 2082, Level 2, Clinical Sciences Corridor, Westmead Hospital, Cnr Darcy and Hawkesbury Roads, Westmead, Sydney, NSW 2145 Australia; 2 Sydney Medical School (Westmead Clinical School), The University of Sydney, Sydney, NSW 2145 Australia; 3 Centre for Vision Research, Westmead Institute for Medical Research, Sydney, NSW Australia 2145; 4 School of Medicine and Public Health, The University of Newcastle, Callaghan, NSW 2308 Australia

**Keywords:** Diabetes mellitus, Heart disease risk factors, Ophthalmoscopes, Surveys and questionnaires, Tonometry

## Abstract

**Aims:**

Endothelial dysfunction is an early risk marker of cardiovascular disease in diabetes mellitus. Timely screening is important in reducing cardiovascular disease-associated morbidity and mortality. This cross-sectional study investigates the acceptability and preferability of non-invasive cardiovascular risk procedures (EndoPAT2000 system and the ECG-gated fundoscope) in participants with diabetes mellitus compared to controls.

**Methods:**

A self-administered Likert scale-based questionnaire was completed by 106 controls and 117 participants with diabetes mellitus, identified through stratified random sampling, upon conclusion of an Australian Heart Eye sub-study conducted at Westmead Hospital, NSW, Australia from 2012 to 2014. Pearson’s *χ*^2^ test, independent-samples t-test and regression analysis were performed.

**Results:**

Study participants who responded to the questionnaire had no preference for procedures (controls: 2.4 ± 1.1 vs diabetes mellitus: 2.5 ± 0.9, *p* = 0.38) but had an overall more negative experience with most aspects of the ECG-gated fundoscope than the EndoPAT2000 system. Of those with diabetes mellitus, participants who provided poorer self-rated health expressed discomfort with the mydriatic drops (ß 0.27, 95%CI 0.001 - 0.54, *p* = 0.049) and the fundoscope’s green light filter (ß 0.27, 95%CI 0.07 - 0.47, *p* = 0.009), as well as maintaining still (ß 0.40, 95%CI 0.08 - 0.72, *p* = 0.02) and not blinking (ß 0.38, 95%CI 0.07 - 0.70, *p* = 0.02) during photo acquisition. These participants were also less willing to repeat the ECG-gated fundoscope procedure (ß 0.29, 95%CI 0.07 - 0.52, *p* = 0.01).

**Conclusions:**

Participants with diabetes mellitus, especially with poorer self-rated health, had a more negative experience with the ECG-gated fundoscope than the EndoPAT2000 system. Difficulties experienced under examination by the ECG-gated fundoscope appear related to the procedural design, which requires amendments improving patient comfort and compliance.

## Introduction

For effective implementation of screening programmes, the principles of screening devised by the World Health Organization should be considered for all screening procedures [1]. The EndoPAT2000 system and digital retinal imaging are two commonly reported non-invasive procedures that are designed to assess endothelial dysfunction for cardiovascular risk screening in different populations. Diabetes mellitus is one population of interest for these procedures because of its strong association with endothelial dysfunction and cardiovascular disease [2]. In a recent study [3], we demonstrated that the ECG-gated fundoscope had a higher test performance than the EndoPAT2000 system at identifying a greater proportion of people with diabetes mellitus with impaired vasoreactivity. However, the designs of both procedures are not without their respective advantages and disadvantages.

The EndoPAT2000 system is designed to examine digital reactive hyperaemia following brachial artery occlusion [4]. This method is a comparable alternative to flow-mediated dilation [5, 6, 7], which is the most accurate non-invasive method for assessing endothelial function. However, unlike flow-mediated dilation [8], it requires minimal training and is less operator dependent. Semi-automated analysis of retinal vessel calibre is another emerging method for cardiovascular risk assessment. Previously, we determined that acquiring digital retinal images at the QRS using an ECG-gated fundoscope improves the accuracy of retinal vessel calibre measurements in controls and diabetes mellitus, by accounting for cardiac cycle-generated pulsatile flow and mechanical part delays [9]. The retina is not subject to autonomic innervation [9] requiring minimal preparation prior to ECG-gated retinal examinations. This is unlike the EndoPAT2000 system that necessitates participants to be fasted and abstain from medications prior to the study [10], which can be difficult in participants with diabetes mellitus. The ECG-gated device can also be easily inserted into the standard fundoscope [11] and provide additional health information, especially for people with diabetes mellitus who are already frequently screened for diabetes-related pathology.

A 2013 Systematic Review [12] reported that a more positive patient experience leads to a more timely diagnosis, quicker clinical decisions and fewer unnecessary referrals or diagnostic tests. Consequently, this leads to improvements in self-rated and objectively measured patient health outcomes. While the EndoPAT2000 system and digital retinal imaging have been developed to improve the assessment of endothelial dysfunction and theoretical patient comfort for cardiovascular risk screening, no study to date has actually investigated the acceptability and preferability of these non-invasive procedures for participants, which is important in determining their usefulness as screening tools. Therefore, this study is the first to compare the preferences and experiences of being examined by the EndoPAT2000 system and the ECG-gated fundoscope in a sample of controls and patients with diabetes mellitus. The results of patient preferences found in this study may subsequently aid in improving the design of both examinations, with the intention of increasing patient comfort. This may then enhance patient compliance, and hence, the quality of data generated from these examinations and patient health outcomes. Thus, this study aims to determine the acceptability and preferability of these cardiovascular risk procedures to people with diabetes mellitus who are at a higher risk of cardiovascular disease.

## Material and methods

### Study participants and ethics approval

The protocol of this cross-sectional study was approved by the Western Sydney Local Health District Human Research Ethics Committee at Westmead Hospital and followed the guidelines of the Declaration of Helsinki. This study’s sample size was based on a previous Australian Heart Eye sub-study conducted by our research group at Westmead Hospital, NSW, Australia, from December 2012 to March 2014. Stratified random sampling was used to recruit participants from the Australian Heart Eye sub-study, of which 106 controls and 117 participants with diabetes mellitus (12 type 1, 105 type 2) responded to the structured questionnaire regarding the experiences and preferences of undergoing non-invasive cardiovascular risk procedures, including the EndoPAT2000 system and the ECG-gated fundoscope (see Appendix). We compared these two groups because one of the groups that these procedures target are people with diabetes mellitus who have a higher risk of cardiovascular disease to the general population. The study sample size was in excess of the 80% power calculation that determined 50 controls and 50 participants with diabetes mellitus were needed to detect a minimum of 1% difference in vascular response between the two groups. All participants provided written informed consent prior to the study. Participants with retinal vascular occlusions, glaucoma, severe cataract, or epilepsy were excluded.

### Data collection and questionnaire

A detailed history was obtained prior to the study including demographic information and past medical history in order to compare the findings of the questionnaire to participants’ self-rated health. Anthropometric measurements such as height(m), weight(kg), and waist circumference(cm) were obtained. Body mass index was calculated as following: weight/height^2^(kg.m^−2^). Cardiovascular measurements such as systolic blood pressure(mmHg) and diastolic blood pressure(mmHg), and heart rate(bpm) were obtained using an electronic blood pressure device (Model HEM-907; OMRON Healthcare, Victoria, Australia). Mean arterial pressure(mmHg) was calculated as following: diastolic blood pressure + 1/3(systolic blood pressure – diastolic blood pressure). Digital macula and optic disc-centred coloured retinal photographs were graded for diabetic retinopathy and maculopathy in accordance with the Modified Airlie House Classification of diabetic retinopathy guidelines [13] and the Wisconsin Age-related Maculopathy Grading System [14], respectively. Eight participants in the control group, later determined to have diabetic retinopathy in either eye, were assumed to have undiagnosed diabetes mellitus and were included in the group with diabetes mellitus.

An inhouse 5-point Likert scale-based questionnaire was distributed to participants who were examined by the EndoPAT2000 system and the ECG-gated fundoscope. Protocols for both procedures are described elsewhere [15]. The questionnaire consisted of 16 questions related to the level of discomfort or difficulty with aspects of each examination, and the duration of examinations. Participants were requested to complete the questionnaire immediately following the procedures to prevent recall bias. Participants were also requested to provide honest opinions of the procedures in order to reduce social desirability bias. The Likert scale transitioned from a highly positive to a very negative experience. Participants were also asked to rate their overall health as poor, fair, good or excellent.

### Statistical analyses

All data were entered, cross checked by two reviewers for errors, and analysed using the Statistical Package for the Social Sciences software (version 26.0 for Macintosh, SPSS Inc., Chicago, Illinois, USA). Only valid data were analysed as missing values were excluded from the analysis. Normality was assessed using the Shapiro-Wilk test with the assistance of box plots for visual inspection. Continuous variables were presented as means and standard deviations; the independent-samples t-test compared mean differences between groups. Categorical variables were presented as frequencies and percentages with significant differences assessed by Pearson’s *χ*^2^ test in samples greater than 5 and Fisher’s exact test in samples less than 5. Bonferroni *χ*^2^ residual analysis was performed on categorical subset groups with a Bonferroni-adjusted *p* value to assess statistical significance. Otherwise, statistical significance for all other analyses was attributed at *p* < 0.05. Multiple linear regression was used to determine associations between study questions and participant characteristics (diabetes mellitus status in all participants and self-health rating in participants with diabetes mellitus only). Apart from these characteristics, the model also included age, sex, diabetic retinopathy status, smoking status, hypertension, hypercholesterolaemia, fatty liver disease, ethnicity, education status, body mass index, waist circumference, mean arterial pressure, systolic blood pressure, diastolic blood pressure and heart rate. These potential confounders were selected based on statistical significance, possible influence on participants’ subjective experience of health and undergoing cardiovascular risk procedures, or due to being known cardiovascular risk factors. Sub-analyses in participants with diabetes mellitus demonstrated that diabetes status had no association with questionnaire responses from these participants. Thus, participants with type 1 and type 2 diabetes mellitus were analysed as one group.

## Results

**Fig. 1:**
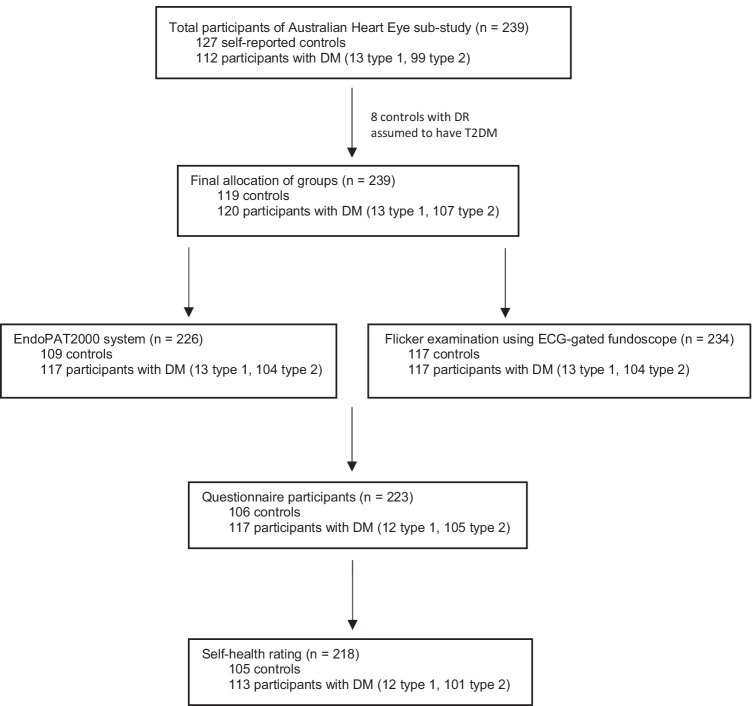
Flowchart of participants from the Australian Heart Eye sub-study who completed the questionnaire. DM, Diabetes Mellitus; DR, Diabetic Retinopathy; T2DM, Type 2 Diabetes Mellitus

**Fig. 2:**
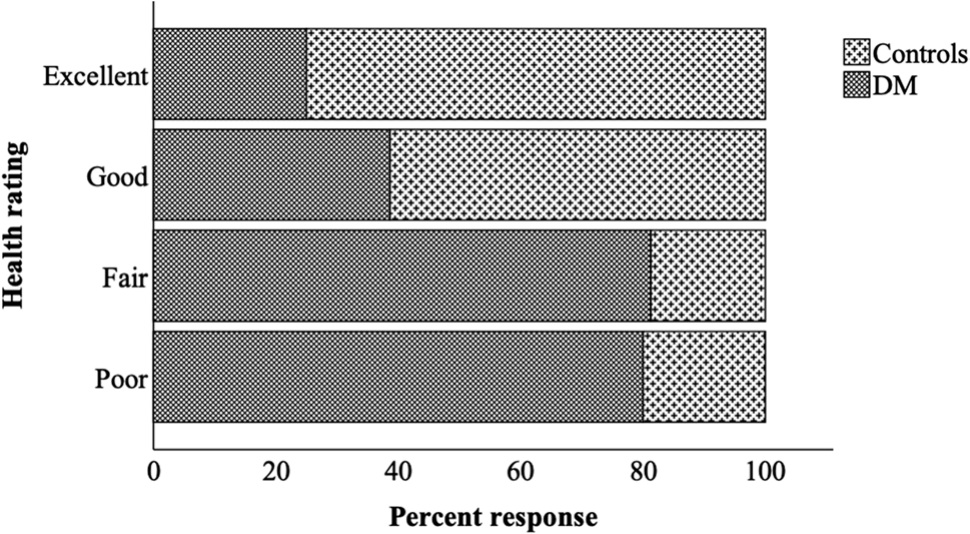
Comparison of self-rated health between controls and participants with diabetes mellitus. DM, Diabetes Mellitus

Of all participants from the Australian Heart and Eye sub-study, 93.3% completed the questionnaire (Figure 1). The main reasons for those who did not complete questionnaires included time constraints or forgetfulness. Participant baseline characteristics are summarised in Table 1. Caucasians and South Asians comprised of the greatest proportion of participants. A higher proportion of South-East Asians and Mediterraneans were controls. A higher proportion of Middle Easterners were participants with diabetes mellitus. Compared to controls, participants with diabetes mellitus were older, and had a higher body mass index, waist circumference, mean arterial pressure, systolic blood pressure, diastolic blood pressure and heart rate. A higher proportion of participants with diabetes mellitus than controls had co-existing hypertension, hypercholesterolemia or fatty liver disease. The majority of controls and participants with diabetes mellitus had no diabetic retinopathy, were never smokers and had a university degree or higher. Pre-existing conditions, including diabetes mellitus, hypertension and hypercholesterolemia, were well controlled by medications. Figure 2 shows the 105 controls and 113 participants with diabetes mellitus of the total questionnaire participants who provided a rating of their health. Of all participants who rated their health as poor or fair, a higher proportion were those who had diabetes mellitus (poor: 80.0% diabetes mellitus, *p* = 0.02; fair: 85.4% diabetes mellitus, *p* < 0.0001). Of all participants who rated their health as good or excellent, a higher proportion were controls (good: 58.0% controls, *p* = 0.001; excellent: 72.2% controls, *p* = 0.001).

**Table 1:** Questionnaire participant baseline characteristics

Variable	Controls (*n* = 106)		DM (*n* = 117)		
	n (%)	Mean (± SD)	n (%)	Mean (± SD)	*p* value
Age (years)	106	40.4 ± 12.6	117	47.8 ± 13.0	<0.0001
Sex	106		117		0.08
Male	42 (39.6)		60 (51.3)		
Female	64 (60.4)		57 (48.7)		
Ethnicity^a,b^	106		117		0.009**
Caucasian	31 (29.2)		37 (31.6)		0.69
South Asian	32 (30.2)		30 (25.6)		0.42
Middle Eastern	8 (7.5)		19 (16.2)		0.046*
South-East Asian	11(10.4)		4 (3.4)		0.04*
Pacific Islander	3 (2.8)		7 (6.0)		0.27
Mediterranean	14 (13.2)		4 (3.4)		0.007**
Mixed race	3 (2.8)		8 (6.8)		0.16
Other	4 (3.8)		8 (6.8)		0.32
Education^a,c^	102		98		<0.0001
No school certificate	1 (1.0)		17 (17.3)		<0.0001
School or intermediate certificate	3 (2.9)		7 (7.1)		0.16
Higher school or leaving certificate	3 (2.9)		13 (13.3)		0.007**
Trade/apprenticeship	3 (2.9)		5 (5.1)		0.42
Certificate/diploma	17 (16.7)		23 (23.5)		0.23
University degree or higher	75 (73.5)		33 (33.7)		<0.0001
Smoking status^a,d^	106		117		0.0005***
Never smoker	84 (79.2)		66 (56.4)		0.0003***
Ex-smoker	20 (18.9)		48 (41.0)		0.0003***
Current smoker	2 (1.9)		3 (2.6)		0.76
Body mass index (kg.m^−2^)	104	25.1 ± 5.9	117	30.4 ± 7.0	<0.0001
Waist circumference (cm)	97	84.9 ± 15.0	111	106.4 ± 21.6	<0.0001
*Blood Pressure (mmHg)*	103		117		
Mean arterial pressure		91.0 ± 9.2		96.1 ± 10.1	<0.0001
Systolic blood pressure		119.4 ± 13.9		127.6 ± 17.0	0.0001***
Diastolic blood pressure		76.8 ± 7.8		80.4 ± 8.2	0.001**
Heart Rate (bpm)	103	69.2 ± 11.1	117	78.7 ± 13.9	<0.0001
*Other Medical Conditions*					
Diabetes duration (years)	–		104	9.4 ± 8.7	–
Hypertension	16 (15.2)		49 (43.0)		<0.0001
Hypercholesterolemia	17 (16.3)		60 (52.2)		<0.0001
Fatty liver disease	2 (1.9)		12 (10.3)		0.008**
*Diabetic Retinopathy*^a,c^	106		117		<0.0001
Nil^e^	102 (96.2)		61 (52.1)		<0.0001
Absent^f^	4 (3.8)		13 (11.1)		0.04*
Questionable^g^	0 (0.0)		5 (4.3)		0.03*
Minimal-mild NPDR	0 (0.0)		20 (17.1)		<0.0001
Moderate-Severe NPDR	0 (0.0)		15 (12.8)		0.0001***
Inactive PDR	0 (0.0)		3 (2.6)		0.09

*DM* Diabetes Mellitus, *NPDR* Non-Proliferative Diabetic Retinopathy, *PDR* Proliferative Diabetic Retinopathy

a*. p* value based on Fisher’s exact test

b. Bonferroni adjusted *p* < 0.003

c. Bonferroni adjusted *p* < 0.004

d. Bonferroni adjusted *p* < 0.008

e. No abnormal changes in retina

f. Vascular changes are related to hypertension rather than diabetes mellitus

g. Microaneurysms absent with either 1) definite hard exudates, soft exudates or intraretinal microvascular abnormalities or 2) definite haemorrhages

*p < 0.05, **p < 0.01, ***p < 0.001

Table 2 presents the unadjusted results of the Likert scale-based questionnaire related to the experiences and preferences for the EndoPAT2000 system and the ECG-gated fundoscope. The mean responses from 117 participants with diabetes mellitus were on average more positive than the 106 controls. There were no significant differences between the two groups in preference for the EndoPAT2000 system compared with the ECG-gated fundoscope (*p* = 0.38). Table 3 outlines statistically significant associations between study questions with diabetes mellitus status in all questionnaire participants, and self-rated health in participants with diabetes mellitus only, after adjusting for confounders. When requested to answer questions specifically about the EndoPAT2000 system, participants with diabetes mellitus experienced less difficulty lying still for 15 minutes compared with controls. Although both groups were inclined to repeat the examination in the future if required, participants with diabetes mellitus were more willing than controls. Participants were also requested to answer questions based on the ECG-gated fundoscope. Both groups experienced a moderate level of difficulty in maintaining still during photograph acquisition (*p* = 0.65). Otherwise, participants with diabetes mellitus generally had less issues with this procedure than controls, including experiencing less discomfort in the application of mydriatic drops, and on exposure to the red light, green light and flickering light. Participants with diabetes mellitus also experienced less difficulty with not blinking during photograph acquisition than controls. Although both groups felt it was easier to prepare for a camera flash with the study coordinator’s guidance, participants with diabetes mellitus found it more beneficial than controls. Participants with diabetes mellitus also rated the duration of the flickering light retinal examination as more acceptable than controls and were more willing to repeat the retinal examination in the future, if required, than controls. However, poorer self-rated health in participants with diabetes mellitus was associated with greater discomfort from the eye drops and green light filter, and a greater difficulty in maintaining still and not blinking during photo acquisition. Of all participants with diabetes mellitus, those individuals with poorer self-rated health were also less inclined to repeat the ECG-gated fundoscope procedure.

**Table 2:** Questionnaire responses from controls and participants with diabetes mellitus of being examined by EndoPAT2000 system and ECG-gated fundoscope

	Mean Score (SD)		
Question^a^	Controls *n* = 106	DM *n* = 117	*p* value
Do you prefer the EndoPAT or retinal photographs more?			
*1 = EndoPAT2000* *5 = Retinal photographs*	2.4 (1.1)	2.5 (0.9)	0.38
EndoPAT2000 system questions			
What level of difficulty did you experience fasting?			
*1 = No difficulty* *5 = High difficulty*	1.6 (1.1)	1.3 (0.7)	0.03*
What degree of discomfort did you experience during the 5 minute compression around the arm?			
*1 = No discomfort* *5 = High discomfort*	2.4 (1.1)	2.0 (1.2)	0.02*
How difficult was it lying still for 15 minutes?			
*1 = No difficulty* *5 = High difficulty*	1.9 (1.0)	1.4 (0.9)	0.0005***
Would you have the EndoPAT test performed again?			
*1 = Yes definitely* *5 = Definitely not*	1.7 (1.0)	1.3 (0.8)	0.0005***
ECG-gated fundoscope questions			
What degree of discomfort did the eyedrops provide?			
*1 = No discomfort* *5 = High discomfort*	2.6 (0.9)	2.4 (1.1)	0.049*
How difficult was it to maintain still during retinal photograph acquisition?			
*1 = No difficulty* *5 = High difficulty*	2.5 (1.2)	2.5 (1.3)	0.65
How difficult was it not to blink during photo acquisition?			
*1 = No difficulty* *5 = High difficulty*	3.1 (1.3)	2.6 (1.3)	0.003**
What degree of discomfort did the flash provide?			
*1 = No discomfort* *5 = High discomfort*	3.1 (1.1)	2.5 (1.3)	0.0002***
Did it make it easier to prepare for when the camera flashed with the study coordinator’s guidance?			
*1 = Yes definitely* *2 = Definitely not*	1.5 (0.9)	1.1 (0.5)	0.0001***
What degree of discomfort did the green light provide?			
*1 = No discomfort* *5 = High discomfort*	2.3 (1.0)	1.6 (0.8)	<0.0001
What degree of discomfort did the red light provide?			
*1 = No discomfort* *5 = High discomfort*	2.7 (1.1)	2.3 (1.2)	0.02*
What degree of discomfort did the flickering light provide?			
*1 = No discomfort* *5 = High discomfort*	2.7 (1.2)	1.9 (1.1)^b^	<0.0001
How would you rate the duration of the flicker test?			
*1 = Acceptable* *5 = Too long*	2.4 (1.3)^c^	1.9 (1.1)^b^	0.001**
Did you feel reasonably well 5 hours post retinal photographs?			
*1 = Yes definitely* *5 = Definitely not*	1.7 (1.1)	1.3 (0.8)	0.001**
Would you do the eye test again?			
*1 = Yes definitely* *5 = Definitely not*	2.2 (1.3)	1.5 (1.0)	<0.0001

*DM* Diabetes Mellitus

a. All responses are based on a total of 5

b. Valid *n* = 115

c. Valid *n* = 105

*p < 0.05, **p < 0.01, ***p < 0.001

**Table 3:** Multiple linear regression analyses of the association between examination questions and participant characteristics

	Question	ß	95%CI	*p* value
All participants				
DM	How difficult was it lying still for 15 minutes?	−0.49	−0.79 - -0.19	0.001**
	Would you have the EndoPAT test performed again?^a^	−0.29	−0.57 - -0.01	0.04*
	What degree of discomfort did the eyedrops provide?	−0.34	−0.63 - -0.05	0.02*
	How difficult was it not to blink during photo acquisition?^b^	−0.68	−1.09 - -0.26	0.001**
	Did it make it easier to prepare for when the camera flashed with the study coordinator’s guidance?	−0.38	−0.60 - -0.17	0.0006***
	What degree of discomfort did the green light provide?	−0.76	−1.02 - -0.49	<0.0001
	What degree of discomfort did the red light provide?	−0.46	−0.79 - -0.13	0.007**
	What degree of discomfort did the flickering light provide?^c^	−0.76	−1.09 - -0.44	<0.0001
	How would you rate the duration of the flicker test?^d^	−0.45	−0.80 - -0.11	0.01*
	Would you do the eye test again?^e^	−0.61	−0.97 - -0.26	0.0009***
DM				
	What level of difficulty did you experience fasting?^f^	0.18	0.01 - 0.34	0.04*
Self-rated health	What degree of discomfort did the eyedrops provide?	0.27	0.001 - 0.54	0.049*
	How difficult was it to maintain still during retinal photograph acquisition?^g^	0.40	0.08 - 0.72	0.02*
	How difficult was it not to blink during photo acquisition?^b^	0.38	0.07 - 0.70	0.02*
	What degree of discomfort did the green light provide?	0.27	0.07 - 0.47	0.009**
	How would you rate the duration of the flicker test?^d^	0.39	0.15 - 0.64	0.002**
	Would you do the eye test again?^d^	0.29	0.07 - 0.52	0.01*

Independent variables included in the model were: self-rated health, age, sex, diabetic retinopathy status, smoking status, hypertension, hypercholesterolaemia, fatty liver disease, ethnicity, education status, body mass index, waist circumference, mean arterial pressure, systolic blood pressure, diastolic blood pressure and heart rate

*DM* Diabetes Mellitus

a. Adjusted for hypercholesterolaemia and smoking status

b. Adjusted for age and self-rated health

c. Adjusted for age

d. Adjusted for diastolic blood pressure

e. Adjusted for hypercholesterolaemia

f. Adjusted for body mass index

g. Adjusted for age and heart rate

*p < 0.05, **p < 0.01, *** *p* < 0.001

## Discussion

The correlation between diabetes mellitus and vasoreactivity measured by non-invasive cardiovascular risk procedures, including the EndoPAT2000 system and digital fundoscopy, have been explored widely. However, in order for these screening procedures to be successfully implemented, their acceptability to their target population needs to be determined. This is the only study at present to explore the acceptability and preferability of using the EndoPAT2000 system and the ECG-gated fundoscope in participants with diabetes mellitus compared with controls. Both groups did not prefer either of the two procedures. Participants with diabetes mellitus on average had a more positive response of using both procedures and were more willing to repeat them, compared with controls. However, participants with diabetes mellitus with poorer self-rated health had a more negative experience under examination of the ECG-gated fundoscope than the EndoPAT2000 system, and were less willing to repeat this procedure in the future.

Both groups experienced difficulties with most aspects of the ECG-gated fundoscope procedure. While participants with diabetes mellitus are generally known to be more conditioned for retinal photography, difficulties with the examination persisted in this group, especially in those with poorer self-rated health. There are various issues behind the process of retinal photography. People who are photosensitive are also more prone to blinking during photography, which can introduce blink artefacts, such as eyelashes, that obscure the captured image [16]. Mydriasis can provide discomfort, increase examination time, and prevent people from driving for some time post-application [17, 18]. Although not observed in this study’s participants, there is a small risk for mydriasis-induced acute angle-closure glaucoma, especially in ethnically susceptible populations such as Asians due to differing ocular anatomy [19]. Pupil dilation can also impair lens accommodation [20], resulting in blurred vision and affecting the ability to focus on the fixation light when capturing optic disc-centralised photographs. However, when pupils are not dilated, time delays of 5-7 min are required for capturing serial images so that the pupils have adequate time to recover from the effects of the camera flash [21]. The camera flash is unavoidable in many circumstances, especially with darker irises, in order to adequately illuminate and visualise the retina.

The difficulty that many participants experienced in maintaining open eyes during image capture can be explained by the dazzle reflex [22]. This is a primitive reflex of the human visual system under dim conditions akin to the environment in which photo acquisition occurred in our study. It triggers ocular spasms to reduce the discomfort and pain associated with the intensity of bright lights on the retina [23]. Thus, the suppression of this reflex can be difficult to achieve but appears to be easier when the photographer has prepared the participant for a camera flash. Dilating pupils can also become difficult due to diminishing pupil size with age, uncontrolled diabetes or increased diabetes duration [24, 25], and darker irises [26]. However, gradable quality images in many of these individuals are only possible to obtain by mydriasis. Mydriasis has shown to reduce the proportion of ungradable photographs even though it does not improve the sensitivity and specificity of detecting retinal pathology [27]. In relation to vessel grading, a dilated pupil improves the resolution of retinal vessel calibre, which enables semi-automated analysis software to track the vessel width more accurately.

Red-free photographs also enable accurate width detection because they enhance the contrast of retinal structures, such as the retinal microvasculature, which is difficult to achieve with colour photographs [28]. This is because removing red light, normally reflected from haemoglobin, can darken the appearance of blood vessels [29]. Both groups in this study also experienced the green light to be less intense and therefore more tolerable than the red filter, which is another advantage of this technique. However, flickering green light was less tolerable than constant green light, which could be due to sensory overload and visual discomfort from artificial flickering patterns [30]. Remaining stationary during photo acquisition is also important as this can affect photograph quality and the ability to grade retinal vessels accurately [31]. Small eye movements can change the angle of photo acquisition and therefore influence calibre measurements. Even subtle movements can blur images enough to alter the magnification of blood vessels, making them appear smaller or larger than the actual size.

A clear limitation of this study is its small sample size. However, the major strengths include the minimisation of the participants’ recall bias and its realistic representation of the ethnically diverse groups that seek healthcare in multicultural nations. This study had good internal validity as the responses were adjusted for variables that may have also influenced the responses. The questions in this survey were devised in consultation with a Cardiologist and Ophthalmologist who are both experts in this research area, which ensured that the content validity was optimal in addressing the objectives of this study. The test-retest reliability of this survey is one area that future studies must explore to determine whether the survey results are reproducible across time. In this survey, however, there was good internal consistency of responses to various aspects of each procedure. Furthermore, this study is the first to compare the preferences and experiences of being examined by the EndoPAT2000 system and the ECG-gated fundoscope in a sample of controls and participants with diabetes mellitus. We identified those people with diabetes mellitus who were less likely to routinely engage in retinal examinations and require closer monitoring of their eye health. Further research is still required to determine whether improving the fundoscope design to be more patient-friendly improves patient follow-up in retinal examinations. 

In summary, despite having no particular preference for either procedure, the controls and participants with diabetes mellitus had a more negative experience with the ECG-gated fundoscope than the EndoPAT2000 system. This was more so the case for participants with diabetes mellitus who had poorer self-rated health. Our study highlights the need to improve the design of retinal photography in order to increase participant comfort during examinations, which is important in enhancing participant compliance and the quality of data collected. However, a larger population study is required to consolidate this study’s findings.

## Data Availability

The raw quantitative dataset used to support the findings of this study are deidentified participant data and are available from the corresponding author on reasonable request. Please contact Dr. Anchal Lal: alal2824@alumni.sydney.edu.au if interested.
